# Exercise Pulmonary Hypertension in Heart Valve Disease

**DOI:** 10.31083/j.rcm2504131

**Published:** 2024-04-02

**Authors:** Alessandra Schiavo, Michele Bellino, Antonella Moreo, Francesca Casadei, Andreina Carbone, Salvatore Rega, Rodolfo Citro, Raffaele Sangiuolo, Antonio Cittadini, Eduardo Bossone, Alberto M. Marra

**Affiliations:** ^1^Cardiology and Intensive Coronary Care Unit, Fatebenefratelli Hospital, 80123 Naples, Italy; ^2^Cardio-Thoracic and Vascular Department, University Hospital “San Giovanni di Dio e Ruggid’Aragona”, 84131 Salerno, Italy; ^3^Cardiology IV, “A. De Gasperis” Department, Niguarda Ca' Granda Hospital, 20162 Milan, Italy; ^4^Unit of Cardiology, University of Campania “Luigi Vanvitelli”, 80138 Naples, Italy; ^5^Department of Public Health, University of Naples “Federico II”, 80131 Naples, Italy; ^6^Department of Translational Medical Sciences, “Federico II” University, 80131 Naples, Italy; ^7^Gender Interdepartmental Institute of Research (Genesis), “Federico II” University, 80131 Naples, Italy

**Keywords:** heart valve diseases, exercise stress echocardiography, exercise pulmonary hypertension, right ventricle, right heart

## Abstract

The optimal management of heart valve disease (HVD) is still debated and many studies are underway to identify the best time to refer patients for the most appropriate treatment strategy (either conservative, surgical or transcatheter interventions). Exercise pulmonary hypertension (PH) can be detected during exercise stress echocardiography (ESE) and has been demonstrated to have an important prognostic role in HVD, by predicting symptoms and mortality. This review article aims to provide an overview on the prognostic role of exercise PH in valvulopathies, and its possible role in the diagnostic-therapeutic algorithm for the management of HVD.

## 1. Introduction

**Fig. 1.**
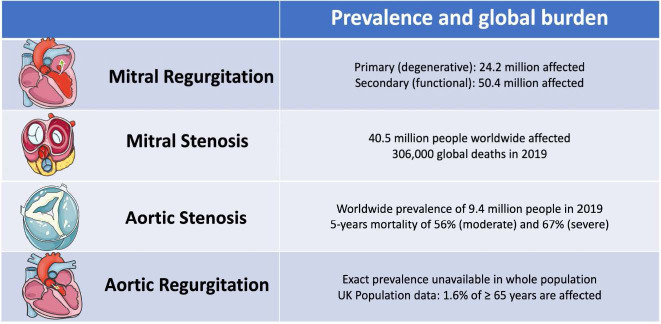
**Prevalence and global burden of left heart valve diseases (adapted from Coffey S *et al*. [1], “Global epidemiology of valve heart disease”)**.

Heart valve diseases (HVD) is a common etiology of heart failure, which is a prominent driver of hospitalization and mortality in cardiovascular disease (Fig. 1, Ref. [1]). Echocardiography is the gold standard technique for the diagnosis of HVD, assessing mechanisms and severity of valve disease and guiding the clinician to select the most appropriate treatment strategy. Clinical presentation and symptomatic status are pivotal to plan treatment strategy, but prognosis and therapeutic management are mainly influenced by echocardiographic features, such as left ventricular ejection fraction (LVEF), left ventricular filling pressures, right ventricular function (RVF) and estimation of pulmonary hypertension (PH). PH is commonly associated with HVD, being present either at rest or detected after exertion with stress testing (Fig. 1).

**Fig. 2.**
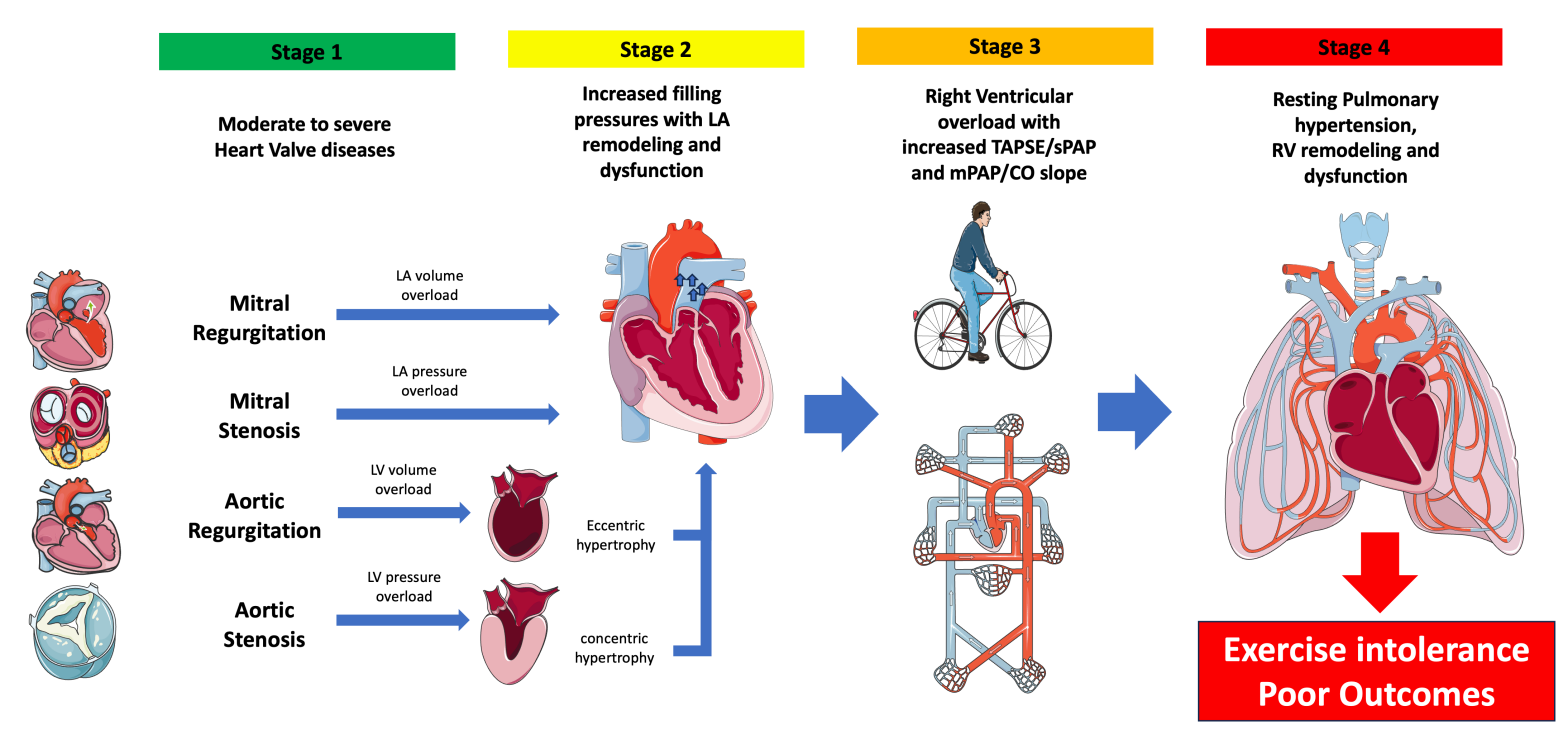
**Key patho-mechanisms of pulmonary hypertension and right ventricular failure in heart valve disease**. LA, left atrium; LV, left ventricle; TAPSE/sPAP, tricuspid annular plain systolic excursion/systolic pulmonary arterial pressure; mPAP/CO, mean pulmonary arterial pressure/cardiac output; RV, right ventricle.

The pathophysiological mechanisms underlying the development of PH in left HVD are summarized in Fig. 2: in mitral regurgitation (MR), left atrium (LA) volume overload and left ventricle (LV) volume overload occurs; in mitral stenosis (MS) mainly an increase in LA pressures and LV filling pressures takes place; again, aortic regurgitation (AR) leads to LV volume and pressure overload, while aortic stenosis (AS) causes an increase in LV pressures (Stage 1). As a consequence, LV remodeling and diastolic dysfunction occurs, resulting in increased filling pressures and LA dilatation and dysfunction (Stage 2). The retrograde transmission of LA pressure to the pulmonary circulation unit leads to a progressive right ventricle (RV) pressure overload (Stage 3), which, finally, results in the development of PH and subsequent RV remodeling and dysfunction (Stage 4).

According to European Society of Cardiology (ESC) PH guidelines [2], PH due to left HVD belongs to group 2, which includes all forms caused by underlying left-sided heart disease. In addition to right heart catheterization, resting echocardiography may also help in assessing the probability of PH: a tricuspid regurgitation velocity (TRV) greater than 3.4 m/s is highly suspicious for PH (class I, level of evidence B) [2]. Additional signs of right heart overload such as right heart enlargement, reduced TAPSE/sPAP (tricuspid annular plain systolic excursion/systolic pulmonary arterial pressure) ratio or a “D-shaped” left ventricle may help make the diagnosis of PH in patients with a TRV between 2.7 and 3.4. PH is indicative of the severity of the underlying valvular disease. PH complicates severe and symptomatic mitral valve disease in 60–70% of patients [3]. Almost half of symptomatic patients with aortic stenosis may present with PH [4]. Detection of exercise-induced PH during a stress-test is critical, since it is indicative of decreased survival, regardless of the presence of symptoms at rest.

Exercise stress echocardiography (ESE) is not only specifically indicated in HVD. However, it could be helpful to assess the origin of stress-related dyspnea during exertion in patients with HVD who are asymptomatic at rest. Therefore, ESE is indicated in those cases with an apparent mismatch between resting echocardiography and patient-reported exercise symptoms [5]. During exercise, haemodynamic changes include increasing of stroke volume and heart rate, with a reduction of systemic vascular resistance resulting in increased systolic pulmonary arterial pressure (sPAP). On exertion, healthy individuals will increase pulmonary artery pressures proportional to the increase in cardiac output; a slope of the relationship mean PAP/cardiac output >3 mmHg/L/min assessed by exercise right heart catheterization defines exercise PH [2]. Consequently, in HVD, sPAP at exercise also mirrors the rise in LA pressure according to the severity of HVD, LV diastolic dysfunction and RV function [6].

Either direct (mitral regurgitation/stenosis) as well as indirect (aortic regurgitation/stenosis) exposure of the LA to pressure overload may trigger remodeling characterized by LA dilatation, interstitial fibrosis and systolic/diastolic failure [7]. Once the LA has lost its compliance and reservoir function, there is an increase in LV filling pressure [8, 9] which becomes more evident during exercise. The development of PH in the context of HVD is not only attributed to a mere retrograde transmission of hemodynamic pressures to the right-heart circulation unit. Superimposed mechanisms of pulmonary vascular remodeling due to capillary damage with alveolar edema may lead to collagen deposition and fibrosis, more so in the pulmonary veins, with lesions similar to those observed in pulmonary veno-occlusive disease [6].

A cut-off of sPAP >60 mmHg measured with echocardiography at rest has been found to be a marker for poor outcomes in HVD. Nevertheless, stress echocardiography and exercise PH do not have a well-defined role within the diagnostic-therapeutic flow chart for clinical management of HVD.

## 2. Mitral Valve

### 2.1 Mitral Regurgitation

#### 2.1.1 Resting PH – Prevalence and Prognosis 

Mitral regurgitation is one of the most common HVD linked with PH at rest. The prevalence of PH depends on the presence of symptoms, the grade of MR severity and the LV systolic function. In primary MR, the prevalence of PH varies from 20–30% in symptomatic patients and from 6 to 30% in asymptomatic patients. The presence of resting PH >50 mmHg correlates with poor prognosis in patients undergoing mitral valve (MV) surgery (as summarized in Table 1, Ref. [6, 7, 10, 11, 12, 13, 14, 15, 16, 17, 18]), increasing the likelihood of continued post-operative symptoms by >2 fold. However, early surgery still provides the best opportunity for patients to improve their quality of life in those patients with PH at rest. Ghoreishi M *et al*. [19] found that pre-operative sPAP are able to predict both early as well as late survival after surgery. They stress that the optimal timing for surgery should precede the onset of resting PH, defined as a sPAP of 40 mmHg or higher. Patients with severe chronic primary MR with concomitant PH at rest have a worse prognosis after MV repair (especially those <65 years old) with a higher risk of mitral re-operation [10]. According to international recommendations [20, 21], a sPAP value >50 mmHg should be considered as a red-flag to consider mitral valve repair in patients with severe degenerative MR without overt LV dysfunction or dilation (class IIa).

**Table 1.** **Prevalence and prognostic role of rest PH and exercise PH in left heart valve disease**.

		Rest PH (sPAP >50 mmHg)		Exercise PH (sPAP >60 mmHg)	
	Clinical Status	Prevalence	Prognosis	Prevalence	Prognosis
Aortic Stenosis	Symptomatic	15–30%	- In 14,980 patients, risk of long-term mortality progressively rose as resting sPAP level increased (HR 1.14–2.94, *p* < 0.0001) [17].	-	-
	Asymptomatic	6%	- In 2588 patients, residual PH after TAVR identify patients at increased mortality [16].	55%	- In 69 patients, exercise PH was independently associated with ≈ 2-fold increased risk of cardiac event at almost 2 years follow-up [11].
Aortic Regurgitation	Long-standing Asymptomatic	16–24%	- In 8392 patients, long-term mortality rose as eRVSP increased (aHR 3.32, 95% CI 2.85 to 3.86 in severe PH, *p* < 0.0001) [18].	-	-
Mitral Stenosis	Long-standing Asymptomatic	14–33%	≈3-fold increased risk of death at 10 y (HR 2.98, 95% CI, 1.55–5.75, *p* = 0.001) [14].	>30%	- In 130 patients, sPAP achieved at peak exercise was an important predictor of adverse outcome (aHR 1.025; 95% CI, 1.010–1.040, *p* = 0.001) [15].
Primary Mitral Regurgitation	Symptomatic	20–30%	>2-fold augmented risk of postoperative death [12].	-	-
	Asymptomatic	6–30%	- Between 382 patients with asymptomatic severe degenerative MR undergoing MV repair, those with PH displayed a doubled-risk of late mortality compared with the remaining patients (HR 2.54; 95% CI, 1.17–4.80, *p* = 0.018) [7].	≈ 50%	- In 78 patients, exercise PH (but not resting PH) was independently associated with the occurrence of symptoms (HR = 3.4, *p *= 0.002) [10].
Secondary Mitral Regurgitation	Symptomatic for most	37–62%	- In 873 patients, operative mortality was correlated with the degree of preoperative PH (2%, 3%, 8%, and 12% for none, mild, moderate, and severe PH, respectively, *p* < 0.0001) [6].	40%	- In 159 patients, incidence of cardiac events during follow-up was significantly higher in patients with exercise PH compared with those without exercise PH (4 years: 40 ± 7% *vs*. 20 ± 5%, *p* < 0.0001) [13].

Adapted from Filippetti L *et al*. [14], “The Right Heart Pulmonary Circulation Unit and Left Heart Valve Disease”. PH, pulmonary hypertension; sPAP, systolic pulmonary arterial hypertension; CI, confidence interval; eRVSP, estimated right ventricle systolic pressure; HR, hazard ratio; aHR, adjusted hazard ratio; TAVR, transcatheter aortic valve replacement; MR, mitral regurgitation; MV, mitral valve.

In cases of secondary MR, which are usually symptomatic, the prevalence of resting PH ranges from 37 to 62% of patients and is a known independent predictive factor for chronic heart failure or death from any cause. According to this report, mortality increases as PH rises, further supporting the role of PH in predicting poor outcomes [22].

#### 2.1.2 Exercise PH – Prevalence and Prognosis 

In asymptomatic patients with severe primary MR, exercise PH is more commonly found than resting PH, with a prevalence of about 50% of patients (see Table 1). Exercise-PH correlates with a >3-fold increased risk of recurrent symptoms and predicts the recurrence of symptoms better than resting PH [23]. Moreover, exercise-PH is associated with significantly lower 2-year symptom-free survival. When stress PH is greater than 60 mmHg, patients should be considered for surgery [14]. In a cohort of 97 patients with moderate-severe primary MR, PH at peak-exercise was a predictor of the recurrence of symptoms within 2 years [24]. Exercise-PH was estimated to be present in 40% [14] of patients with functional MR regardless of LV function. The independent determinants of exercise-PH are the occurrence of resting PH, MR severity with exercise, alongside with left ventricular diastolic dysfunction [13]. Exercise PH is associated with poor outcomes, regardless of the degree of the severity of secondary MR, and results in a 5.3-fold increase risk for cardiac death due to cardiovascular causes [13].

#### 2.1.3 Role of Stress Echocardiography 

**Fig. 3.**
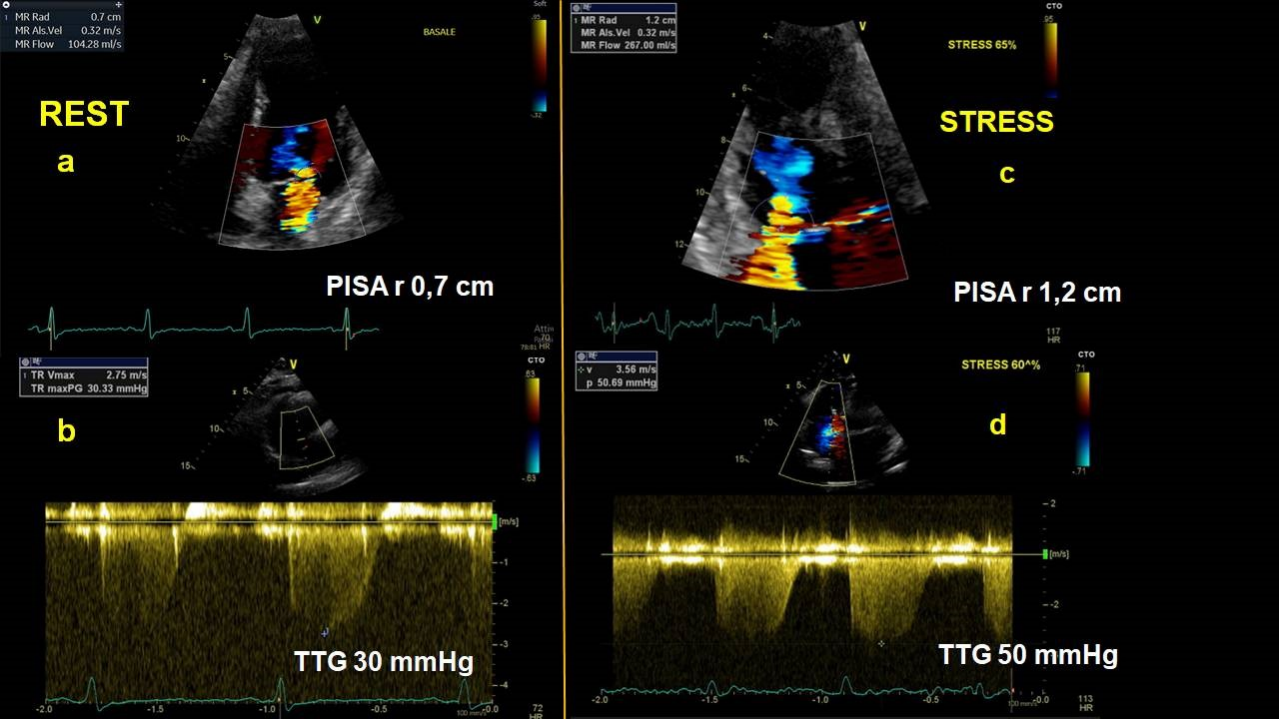
**Mitral regurgitation**. Stress echocardiography in a patient affected by moderate resting mitral regurgitation, showing a dynamic rise in functional mitral regurgitation severity and systolic pulmonary artery pressure at peak of exercise. At rest, PISA-radius is 0.7 cm (panel a) and systolic trans-tricuspid gradient is 30 mmHg (panel b). During exercise, PISA-radius increased up to 1.2 cm (panel c) and trans-tricuspid gradient to 50 mmHg (panel d). PISA-r, Proximal Isovelocity Surface Area-radius; TTG, trans-tricuspid gradient.

ESE may be useful when a mismatch between symptoms and the severity of regurgitation is present at rest [20]. Stress-echo may also help when a mild to moderate resting MR is associated with changes in mitral regurgitant volume and increased sPAP at peak exercise [25] (Fig. 3). Furthermore, ESE may help to assess myocardial reserve contractility [5]. The occurrence of shortness of breath during exercise with a concomitant increase in MR and sPAP identifies a cluster of patients who will benefit take from a tailored therapeutic approach. Current HVD guidelines [20] recommend surgery in asymptomatic patients at rest when severe degenerative MR coexists with preserved LVEF and when exercise PH >60 mmHg is present (Class IIa). This recommendation was already present in the previous edition of the ESC Guidelines [21] (see Table 2, Ref. [20, 21]). In patients with degenerative MR, ESE may be helpful to detect a rise in pulmonary arterial wedge pressure at peak of exercise, which is associated with adverse outcomes [15]. Normally, wedge pressure at rest is strongly dependent on preload and is sometimes underestimated at rest. Therefore, ESE is likely to be a powerful technique to unmask PH in MR before it becomes clinically manifest. When exercise PH and exercise limitations are unmasked by ESE, early surgery should be considered [26].

**Table 2.** **Relevance of PH and/or RV function in the therapeutic handling of heart valve disease: a comparison between 2021 *vs*. 2017 ESC guidelines on HVD**.

	Recommendations in 2021 ESC Guidelines (Ref. [20])			Recommendations in 2017 ESC Guidelines (Ref. [21])		
	RV dilatation and/or dysfunction	Resting PH	Exercise-PH	RV dilatation and/or dysfunction	Resting PH	Exercise- PH
Mitral Regurgitation	-	Pulmonary hypertension (sPAP at rest >50 mmHg) as one of the findings in favor of Surgery in patients with severe primary MR without symptoms and with concomitant preserved LV function (class IIa)	-	-	“Pulmonary hypertension (sPAP at rest >50 mmHg)” as one of the findings in favor of Surgery in asymptomatic patients with severe primary MR with conserved LV function (class IIa)	-
Mitral Stenosis	-	- asymptomatic patients with severe MS: pulmonary hypertension (systolic pulmonary pressure >50 mmHg at rest) prompts performing PMC (class IIa)	-	-	- asymptomatic patients: pulmonary hypertension (systolic pulmonary pressure >50 mmHg at rest) indicates PMC (class IIa)	-
		- symptomatic patients: pulmonary hypertension is one of the unfavourable clinical characteristics for PMC			- symptomatic patients: pulmonary hypertension as one of the unfavourable clinical characteristics for PMC	
Aortic Regurgitation	-	-	-	-	-	-
Aortic Stenosis	-	-	-	-	“Severe pulmonary hypertension (systolic pulmonary artery pressure at rest >60 mmHg confirmed by invasive measurement) without other explanation” as one of the findings in favor of SAVR when severe AS coexists with normal EF (class IIa)	-

RV, right ventricle; PH, pulmonary hypertension; ESC, European Society of Cardiology; HVD, heart valve disease; LV, left ventricle; MR, mitral regurgitation; MS, mitral stenosis; PMC, percutaneous mitral commissurotomy; SAVR, surgical aortic valve replacement; AS, aortic stenosis; EF, ejection fraction; sPAP, systolic pulmonary arterial pressure.

In chronic secondary MR, ESE is particularly useful to determine that MR is the etiology of the patient’s dyspnea (class of recommendation 1 for American College of Cardiology/American Heart Association (ACC/AHA) guidelines 2020 [26]), to determine the etiology of the MR etiology, and to determine myocardial viability [5]. The detection of exercise pulmonary hypertension and the appearance of B-lines on chest-X-ray is indicative of pulmonary congestion (estimated by echocardiogram or right heart catheterization) is a contraindication and both findings are associated with increased mortality. ESC Guidelines [20] recommend surgery in cases of moderate to severe chronic MR, in patients with symptoms despite optimal medical therapy and in those patients with indications for coronary artery bypass graft (CABG surgery). When surgery is not indicated, MV transcatheter edge-to-edge repair (TEER) represents an alternative option especially when COAPT criteria are fulfilled [27]. Time for intervention referral is suggested by sPAP value >50 mmHg [20], while a PH at rest >70 mmHg for TEER. In patients with secondary MR, in 200 consecutive patients, exercise PH was found to play a key role in identifying those most likely to benefit from TEER as opposed to conservative medical management [28].

### 2.2 Mitral Stenosis

#### 2.2.1 Resting PH – Prevalence and Prognosis 

Prevalence of resting PH in MS is related to symptomatic status and MS severity varying from 14 to 33% in moderate PH and ranges from 5 to 9.6% in severe PH [14] (see Table 1). MS is likely to be asymptomatic until PH develops and is associated with recurrence of symptoms. In a recent study, Yang B *et al*. [29] reported that resting PH results in a 3-fold increased risk of death at 10 year follow-up [29]. The authors also reported that moderate to severe PH significative impaired post-operative survival after MV surgery [29]. They concluded that since 10 year post-operative survival is inversely associated with pre-operative sPAP, early referral to surgery should be considered to achieve a better prognosis in MS patients [29].

As summarized in Table 2, in patients with significant MS (i.e., valve area <1.5 ${\mathrm{cm}}^{2}$) but without symptoms, the detection of sPAP >50 mmHg at rest can be considered as an indication to perform percutaneous mitral commissurotomy (PMC) (class IIa Recommendation) [20]. Conversely, in symptomatic patients with significant MS, severe PH favors MV surgery over PMC [20].

#### 2.2.2 Exercise PH – Prevalence and Prognosis 

Exercise PH is likely to occur in >30% of patients presenting with significant MS [14] and is considered to be a marker of severity in MS patients. Its presence is influenced by several hemodynamic features such as atrio-ventricular mean pressure gradient, left atrial volume, and right ventricular function. Recently [30], sPAP at peak of exercise has been reported to be a predictor of poor outcomes in patients with MS. Compared to other left HVD, mitral stenosis is characterized by the strongest correlation between increased left atrial pressure, exercise post-capillary pulmonary pressure, and the onset of symptoms.

#### 2.2.3 Role of Stress Echocardiography 

**Fig. 4.**
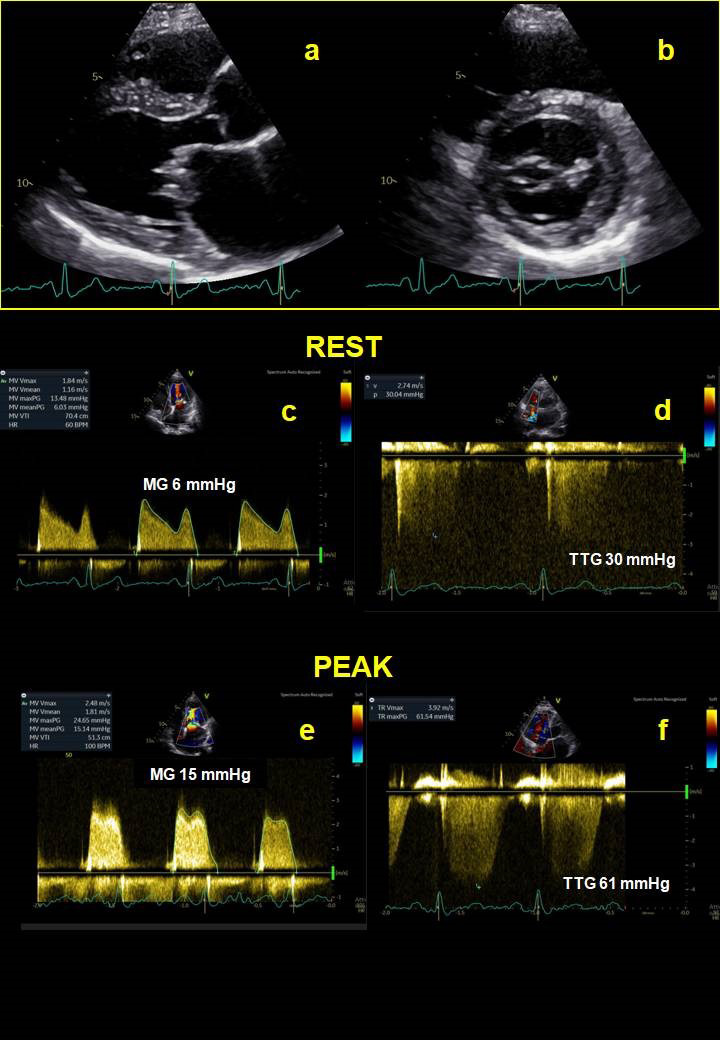
**Mitral stenosis**. Physical stress echocardiography of a 76-year old patient affected by moderate MS at rest. Panel-a and panel-b show a thickened mitral valve with reduced diastolic opening, in 2D long axis parasternal view (a) and in short axis view (b). Panel-c and panel-d show a mean trans-mitral gradient of 6 mmHg and a systolic trans-tricuspid gradient equal to 30 mmHg at rest, respectively. At peak of exercise, mean trans-mitral gradient reaches 15 mmHg (panel-e) and systolic trans-tricuspid gradient increases up to 61 mmHg (panel-f), consistent with significant exercise-PH. MG, mean gradient; TTG, trans-tricuspid gradient.

Stress echocardiography is a helpful tool in patients with MS with a valve area >1.5 ${\mathrm{cm}}^{2}$ and concomitant symptoms. An abnormal response to exercise correlates with either a mean trans-mitral gradient >15 mmHg, or estimated exercise sPAP >60 mmHg [31] (Fig. 4). In these patients, referral for mitral valve commissurotomy should be considered (class II b) [15, 26, 32].

## 3. Aortic Valve

### 3.1 Aortic Stenosis

#### 3.1.1 Resting PH – Prevalence and Prognosis 

AS is the most common HVD in western countries [20]. PH detected by echocardiography in symptomatic patients is found between 15–30% of patients [14, 33]. This value has also been confirmed by studies with right heart catheterization performed before transcatheter aortic valve replacement (TAVI) [16, 34]. In contrast, the prevalence of PH in asymptomatic AS is lower, about 6% as reported in several studies [14, 35] (see also Table 1).

The significance of PH complicating AS remains poorly characterized. A large retrospective study involving 14,980 patients with at least moderate AS, demonstrated that PH negatively affected prognosis even at mildly increased pulmonary pressures with a significant increase in mortality when PH becomes more severe [17].

PH impacts outcomes in symptomatic AS, both in conservative (medical) treatment and after intervention (whether surgical or transcatheter) [16, 34]. A recent metanalysis [36] showed how much baseline PH predicts mortality in patients with severe AS after TAVI. PH is associated with increased long-term cardiac mortality and all-cause mortality, using a resting sPAP cut-off of 60 mmHg or higher. In a recent study performed on 617 consecutive patients with severe AS undergoing TAVI, 46% of the study population experienced a reduction of sPAP after intervention, whereas residual PH resulted in a higher risk of all-cause mortality after 30 days [37].

Less data are available for asymptomatic forms of AS and limited data are also available regarding prognosis and survival. Likewise, little is known about the role played by PH in low-flow, low-gradient AS. This population is unquestionably the most difficult to treat and the timing for intervention is still a very challenging issue.

#### 3.1.2 Exercise PH – Prevalence and Prognosis

**Fig. 5.**
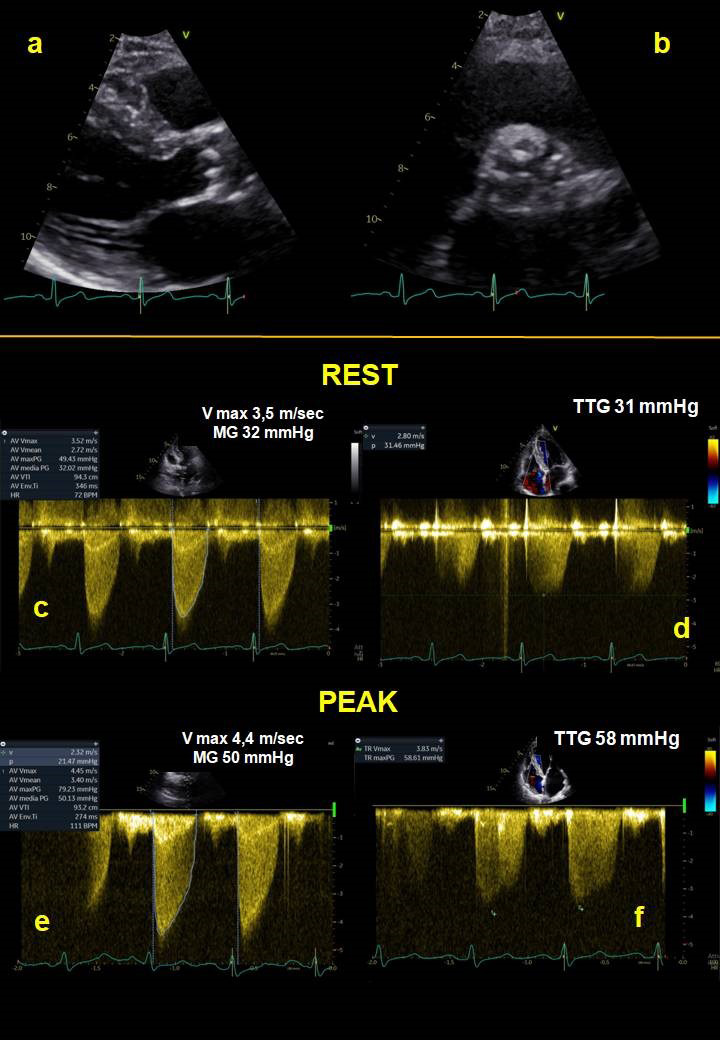
**Aortic stenosis**. Stress-echocardiography in a patient affected by moderate aortic stenosis (AS) at rest. Panel-a and panel-b show a calcific aortic valve with reduced systolic opening in a 2D long axis parasternal view (a) and a short axis view (b). At rest, aortic valve peak velocity is 3.5 m/sec resulting in a mean gradient of 32 mmHg (panel-c), systolic trans-tricuspid gradient of 31 mmHg (panel-d). At peak of exercise, aortic valve peak velocity increases up to 4.4 m/sec with a mean gradient of 50 mmHg (panel-e) and systolic trans-tricuspid gradient reaches 58 mmHg (panel-f), consistent with a significant exercise-PH. AV-Vmax, aortic valve peak velocity; MG, mean gradient; TTG, trans-tricuspid gradient; PH, pulmonary hypertension.

In a study performed by Lancellotti P *et al*. [35], exercise PH (with aPAP cut-off of 60 mmHg) was more common than resting PH (55% vs 6%) in asymptomatic AS patients. The authors also reported an independent association between exercise PH and a 2-fold increase in the risk of cardiac events after a 3-year follow-up (as summarized in Table 1), supporting the importance of exercise PH as an additional prognostic tool over other echocardiographic parameters, to improve risk-stratification in these patients. Thus, the presence of exercise-induced PH may identify a group of patients with a worse prognosis, in which consideration should be given for earlier intervention (Fig. 5). However, this consideration is not discussed in the current guidelines [20].

#### 3.1.3 Role of Stress Echocardiography

ESE should be performed in asymptomatic patients with severe AS. Current 2021 ESC guidelines on HVD [20] suggest low-dose dobutamine (up to 20 mcg, without atropine) for evaluating low-flow, low-gradient aortic stenosis to discriminate “true” from “pseudo-severe” AS. However, the detection of the presence and/or possible worsening of exercise PH is not always a part in discussion regarding therapeutic decision-making for these patients. The previous version of the 2017 ESC Guidelines [21] mentioned sPAP >60 mmHg at rest (confirmed with invasive measurement), as one of the findings in favor of aortic valve replacement (AVR) in asymptomatic severe AS with normal LVEF (class IIa). Furthermore, given that pulmonary congestion continues to be present after stress testing, the evaluation of pulmonary B-lines during the recovery stage of ESE can add further prognostic information, as stated in the comprehensive ABCDEG stress-echo approach [38].

### 3.2 Aortic Regurgitation

#### 3.2.1 Resting PH – Prevalence and Prognosis

PH develops in the most advanced stages during the course of AR, because the LV has been exposed to chronic volume and pressure overload. The prevalence of severe PH >60 mmHg in severe AR patients is less documented and estimated to be about 16–24% [14]. There is limited data regarding the correlation between PH and AR. A recent study [18] investigated moderate to severe AR patients focusing on the association between PH and mortality. This study found that the presence of PH at rest was related to adverse outcomes, even at mildly elevated levels.

#### 3.2.2 Exercise PH – Prevalence and Prognosis 

Data regarding the correlation between exercise PH and AR is limited. Further studies are necessary to investigate whether stress induced-PH could play a potential role in prognostic stratification that could be useful for planning interventions, and providing more prognostic information on follow-up compared to data obtained during an echocardiogram exam.

#### 3.2.3 Role of Stress Echocardiography 

The role of exercise echocardiography in severe AR patients is still under-investigated. Patients with severe AR and low LVEF were examined in a recent study [39]. The results showed that the increase in LVEF of ≥6% during low-dose dobutamine stress-echo was associated with a significant recovery of post-operative LVEF, without any significant association with reduced cardiovascular events. Another recent study [40] evaluated patients with asymptomatic severe AR with preserved ejection fraction (EF) aiming to find a predictive value for stress echocardiography. The study found that the absence of contractility reserve was associated with post-operative deterioration of LV contractility and the occurrence of symptoms [40]. These observations suggest a possible role of stress echo in AR risk stratification to plan the correct timing for surgery, but further studies are needed. ESE might have a role in asymptomatic AR patients when a mismatch between severity of AR and LV size is present (i.e., moderate AR and disproportionately markedly dilated LV especially with early systolic impairment) or with unexpected involvement of right heart chambers.

## 4. Future Directions

Table 1 summarizes key studies reporting data dealing with the assessment of right ventricular-pulmonary circulation functional unit in HVD, at rest and during exercise. According to available literature there is a pivotal prognostic role of exercise PH in HVD. One of the limitations of exercise echocardiography is the lack of a widely accepted standardization.

**Fig. 6.**
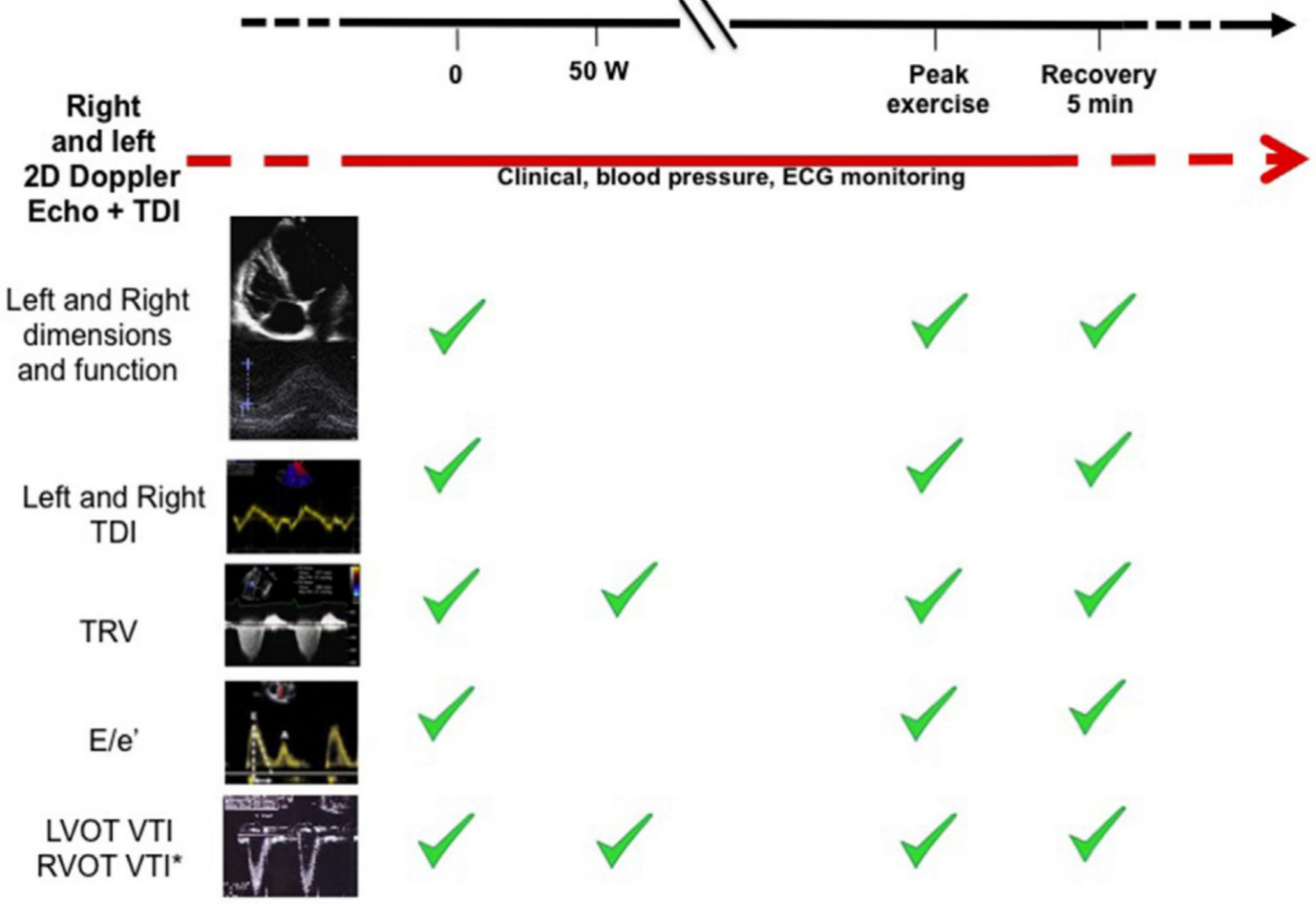
**The RIGHT Heart International NETwork (RIGHT-NET) exercise echocardiography protocol**. (reproduced from Ferrara F *et al*. [42], with license to reuse n. 5627190387). Proposed standardized methodology for exercise stress echocardiography and key echo-Doppler parameters. 2D, 2-Dimensional; TDI, tissue Doppler imaging; TRV, tricuspid regurgitant velocity; E, mitral inflow E velocity as measured by pulse wave Doppler; e’, early diastolic velocity of the lateral and septal (average) mitral annulus as measured by TDI; LVOT, left ventricular outflow tract; RVOT, right ventricular outflow tract; VTI, velocity time integral; ECG, electrocardiography.

The RIGHT-NET Investigators [41, 42] have recently proposed a shared ESE protocol focused on the assessment of right heart and pulmonary circulation (Fig. 6, Ref. [42]). The RIGHT Heart International NETwork (RIGHT-NET) is an international multicenter initiative investigating exercise Doppler echocardiography, focusing on right heart pulmonary circulation, in a wide array of populations including healthy subjects, elite athletes, individuals at risk of or with overt PH [43, 44]. Two studies from the RIGHT-NET cohort have been performed so far, aiming to assess reproducibility [42] and feasibility of exercise echocardiography [45]. Both studies reported a good reproducibility and feasibility with this methodology. A recently published work from the RIGHT-NET investigators reported a strong prognostic value of right ventricular performance under exercise in healthy subjects and in several cardiorespiratory condition [46].

ESE has been conducted with cycloergometer and there are additional protocols using different physical stressors in order to detect exercise PH and RV function. Such protocols may include treadmill [47], handgrip [48] and two-step protocols [49]. Unfortunately, ESE, when specifically focused on right heart-pulmonary circulation unit under stressors, is not included in current guidelines’ recommendations on management of HVD, as shown in Table 2. According to the evidence reported is this review, consideration should be given to the estimation of exercise pulmonary pressures in the diagnostic algorithm of HVD, leading to a better pre-operative risk stratification and providing guidance to achieve the optimal therapeutic strategy. Notwithstanding that the gold-standard for the assessment of pulmonary pressures is still right heart catheterization, ESE represents an affordable, non-invasive diagnostic tool, that is also associated with low complication rates and therefore easily translatable to every day clinical practice as part of a reliable decision-making algorithm.

New evidence from large studies that better analyze this phenomenon is urgently needed.

## 5. Conclusions

HVD are often complicated by both resting as well as exercise PH. While resting PH is considered among factors addressing optimal timing for surgical referral, exercise PH is still underrated in the decision-making process. Through pressure/volume overload, HVD lead to increased LV filling pressure, LA remodeling and dysfunction which are mechanisms resulting in increased pressure to the pulmonary circulation. The ability to adapt to such increases of pulmonary pressure, especially during exercise, is likely to determine the recurrence of symptoms and outcomes following therapeutic interventions. Exercise echocardiography is a reliable non-invasive tool to assess exercise PH. Therefore, there is an urgent need for worldwide standardization and acceptance of this technique.

## Abbreviations

HVD, heart valve diseases; PH, pulmonary hypertension; ESE, exercise stress echocardiography; TRV, tricuspid regurgitation velocity; TAPSE, tricuspid annular plane systolic excursion; sPAP, systolic pulmonary artery hypertension; LV, left ventricle; LVEF, left ventricle ejection fraction; RV, right ventricle; LA, left atrium; MR, mitral regurgitation; MS, mitral stenosis, AS, aortic stenosis; AR, aortic regurgitation; TEER, transcatheter edge-to-edge repair; AVR, aortic valve replacement.

## References

[1] Coffey S, Roberts-Thomson R, Brown A, Carapetis J, Chen M, Enriquez-Sarano M (2021). Global epidemiology of valvular heart disease. *Nature Reviews. Cardiology*.

[2] Humbert M, Kovacs G, Hoeper MM, Badagliacca R, Berger RMF, Brida M (2022). 2022 ESC/ERS Guidelines for the diagnosis and treatment of pulmonary hypertension. *European Heart Journal*.

[3] Tichelbäcker T, Dumitrescu D, Gerhardt F, Stern D, Wissmüller M, Adam M (2019). Pulmonary hypertension and valvular heart disease. *Herz*.

[4] Weber L, Rickli H, Haager PK, Joerg L, Weilenmann D, Brenner R (2019). Haemodynamic mechanisms and long-term prognostic impact of pulmonary hypertension in patients with severe aortic stenosis undergoing valve replacement. *European Journal of Heart Failure*.

[5] Citro R, Bursi F, Bellino M, Picano E (2022). The Role of Stress Echocardiography in Valvular Heart Disease. *Current Cardiology Reports*.

[6] Vachiéry JL, Adir Y, Barberà JA, Champion H, Coghlan JG, Cottin V (2013). Pulmonary hypertension due to left heart diseases. *Journal of the American College of Cardiology*.

[7] Rosenkranz S, Gibbs JSR, Wachter R, De Marco T, Vonk-Noordegraaf A, Vachiéry JL (2016). Left ventricular heart failure and pulmonary hypertension. *European Heart Journal*.

[8] Rossi A, Gheorghiade M, Triposkiadis F, Solomon SD, Pieske B, Butler J (2014). Left atrium in heart failure with preserved ejection fraction: structure, function, and significance. *Circulation. Heart Failure*.

[9] Melenovsky V, Hwang SJ, Redfield MM, Zakeri R, Lin G, Borlaug BA (2015). Left atrial remodeling and function in advanced heart failure with preserved or reduced ejection fraction. *Circulation. Heart Failure*.

[10] Coutinho GF, Garcia AL, Correia PM, Branco C, Antunes MJ (2014). Long-term follow-up of asymptomatic or mildly symptomatic patients with severe degenerative mitral regurgitation and preserved left ventricular function. *The Journal of Thoracic and Cardiovascular Surgery*.

[11] Lancellotti P, Lebois F, Simon M, Tombeux C, Chauvel C, Pierard LA (2005). Prognostic importance of quantitative exercise Doppler echocardiography in asymptomatic valvular aortic stenosis. *Circulation*.

[12] Le Tourneau T, Richardson M, Juthier F, Modine T, Fayad G, Polge AS (2010). Echocardiography predictors and prognostic value of pulmonary artery systolic pressure in chronic organic mitral regurgitation. *Heart (British Cardiac Society)*.

[13] Lancellotti P, Magne J, Dulgheru R, Ancion A, Martinez C, Piérard LA (2015). Clinical significance of exercise pulmonary hypertension in secondary mitral regurgitation. *The American Journal of Cardiology*.

[14] Filippetti L, Voilliot D, Bellino M, Citro R, Go YY, Lancellotti P (2018). The Right Heart-Pulmonary Circulation Unit and Left Heart Valve Disease. *Heart Failure Clinics*.

[15] Barbieri A, Mantovani F (2023). Exercise Stress Echocardiography in Asymptomatic Patients with Severe Primary Mitral Regurgitation: Time to Stepping Out Our Comfort Zone. *The American Journal of Cardiology*.

[16] Miyamoto J, Ohno Y, Kamioka N, Ikari Y, Otsuka T, Tada N (2022). Impact of periprocedural pulmonary hypertension on outcomes after transcatheter aortic valve replacement. *Journal of the American College of Cardiology*.

[17] Ratwatte S, Stewart S, Strange G, Playford D, Celermajer DS (2023). Prevalence of pulmonary hypertension in aortic stenosis and its influence on outcomes. *Heart (British Cardiac Society)*.

[18] Ratwatte S, Playford D, Stewart S, Strange G, Celermajer DS (2023). Prevalence of pulmonary hypertension in aortic regurgitation and its influence on outcomes. *Heart (British Cardiac Society)*.

[19] Ghoreishi M, Evans CF, DeFilippi CR, Hobbs G, Young CA, Griffith BP (2011). Pulmonary hypertension adversely affects short- and long-term survival after mitral valve operation for mitral regurgitation: implications for timing of surgery. *The Journal of Thoracic and Cardiovascular Surgery*.

[20] Vahanian A, Beyersdorf F, Praz F, Milojevic M, Baldus S, Bauersachs J (2022). 2021 ESC/EACTS Guidelines for the management of valvular heart disease: Developed by the Task Force for the management of valvular heart disease of the European Society of Cardiology (ESC) and the European Association for Cardio-Thoracic Surgery (EACTS). *European Heart Journal*.

[21] Baumgartner H, Falk V, Bax JJ, De Bonis M, Hamm C, Holm PJ (2017). 2017 ESC/EACTS Guidelines for the management of valvular heart disease. *European Heart Journal*.

[22] Ratwatte S, Strange G, Playford D, Stewart S, Celermajer DS (2023). Prevalence of pulmonary hypertension in mitral regurgitation and its influence on outcomes. *Open Heart*.

[23] Magne J, Lancellotti P, Piérard LA (2010). Exercise pulmonary hypertension in asymptomatic degenerative mitral regurgitation. *Circulation*.

[24] Althunayyan A, Alborikan S, Badiani S, Wong K, Uppal R, Patel N (2023). Clinical and Prognostic Implications of Cardiopulmonary Exercise Stress Echocardiography in Asymptomatic Degenerative Mitral Regurgitation. *The American Journal of Cardiology*.

[25] Piérard LA, Lancellotti P (2004). The role of ischemic mitral regurgitation in the pathogenesis of acute pulmonary edema. *The New England Journal of Medicine*.

[26] Otto CM, Nishimura RA, Bonow RO, Carabello BA, Erwin JP, Gentile F (2021). 2020 ACC/AHA Guideline for the Management of Patients with Valvular Heart Disease: A Report of the American College of Cardiology/American Heart Association Joint Committee on Clinical Practice Guidelines. *Circulation*.

[27] Adamo M, Fiorelli F, Melica B, D’Ortona R, Lupi L, Giannini C (2021). COAPT-Like Profile Predicts Long-Term Outcomes in Patients with Secondary Mitral Regurgitation Undergoing MitraClip Implantation. *JACC. Cardiovascular Interventions*.

[28] Izumo M, Kuwata S, Ishibashi Y, Suzuki T, Ohara H, Watanabe M (2021). Prognostic impact of transcatheter mitral valve repair in patients with exercise-induced secondary mitral regurgitation. *European Heart Journal. Cardiovascular Imaging*.

[29] Yang B, DeBenedictus C, Watt T, Farley S, Salita A, Hornsby W (2016). The impact of concomitant pulmonary hypertension on early and late outcomes following surgery for mitral stenosis. *The Journal of Thoracic and Cardiovascular Surgery*.

[30] Cristina de Castro Faria S, Costa HS, Hung J, Gorle de Miranda Chaves A, Paes de Oliveira FA, Padilha da Silva JL (2020). Pulmonary Artery Systolic Pressure Response to Exercise in Patients with Rheumatic Mitral Stenosis: Determinants and Prognostic Value. *Journal of the American Society of Echocardiography: Official Publication of the American Society of Echocardiography*.

[31] Lancellotti P, Pellikka PA, Budts W, Chaudhry FA, Donal E, Dulgheru R (2017). The Clinical Use of Stress Echocardiography in Non-Ischaemic Heart Disease: Recommendations from the European Association of Cardiovascular Imaging and the American Society of Echocardiography. *Journal of the American Society of Echocardiography: Official Publication of the American Society of Echocardiography*.

[32] Otto CM, Nishimura RA, Bonow RO, Carabello BA, Erwin JP, Writing Committee Members (2021). 2020 ACC/AHA guideline for the management of patients with valvular heart disease: A report of the American College of Cardiology/American Heart Association Joint Committee on Clinical Practice Guidelines. *The Journal of Thoracic and Cardiovascular Surgery*.

[33] Martinez C, Bernard A, Dulgheru R, Incarnato P, Oury C, Lancellotti P (2016). Pulmonary Hypertension in Aortic Stenosis and Mitral Regurgitation: Rest and Exercise Echocardiography Significance. *Progress in Cardiovascular Diseases*.

[34] Pai RG, Varadarajan P, Kapoor N, Bansal RC (2007). Aortic valve replacement improves survival in severe aortic stenosis associated with severe pulmonary hypertension. *The Annals of Thoracic Surgery*.

[35] Lancellotti P, Magne J, Donal E, O’Connor K, Dulgheru R, Rosca M (2012). Determinants and prognostic significance of exercise pulmonary hypertension in asymptomatic severe aortic stenosis. *Circulation*.

[36] Kokkinidis DG, Papanastasiou CA, Jonnalagadda AK, Oikonomou EK, Theochari CA, Palaiodimos L (2018). The predictive value of baseline pulmonary hypertension in early and long term cardiac and all-cause mortality after transcatheter aortic valve implantation for patients with severe aortic valve stenosis: A systematic review and meta-analysis. *Cardiovascular Revascularization Medicine: Including Molecular Interventions*.

[37] Alushi B, Beckhoff F, Leistner D, Franz M, Reinthaler M, Stähli BE (2019). Pulmonary hypertension in patients with severe aortic stenosis: prognostic impact after transcatheter aortic valve replacement: pulmonary hypertension in patients undergoing TAVR. *JACC: Cardiovascular Imaging*.

[38] Ciampi Q, Cortigiani L, Rivadeneira Ruiz M, Barbieri A, Manganelli F, Mori F (2023). ABCDEG Stress Echocardiography in Aortic Stenosis. *Diagnostics (Basel, Switzerland)*.

[39] Saito C, Arai K, Ashihara K, Niinami H, Hagiwara N (2022). Utility of dobutamine stress echocardiography in aortic valve regurgitation and reduced left ventricular function. *Echocardiography (Mount Kisco, N.Y.)*.

[40] Lee SY, Park SJ, Kim EK, Chang SA, Lee SC, Ahn JH (2019). Predictive value of exercise stress echocardiography in asymptomatic patients with severe aortic regurgitation and preserved left ventricular systolic function without LV dilatation. *The International Journal of Cardiovascular Imaging*.

[41] Ferrara F, Gargani L, Armstrong WF, Agoston G, Cittadini A, Citro R (2018). The Right Heart International Network (RIGHT-NET): Rationale, Objectives, Methodology, and Clinical Implications. *Heart Failure Clinics*.

[42] Ferrara F, Gargani L, Contaldi C, Agoston G, Argiento P, Armstrong WF (2021). A multicentric quality-control study of exercise Doppler echocardiography of the right heart and the pulmonary circulation. The RIGHT Heart International NETwork (RIGHT-NET). *Cardiovascular Ultrasound*.

[43] Vriz O, Veldman G, Gargani L, Ferrara F, Frumento P, D’Alto M (2021). Age-changes in right ventricular function-pulmonary circulation coupling: from pediatric to adult stage in 1899 healthy subjects. The RIGHT Heart International NETwork (RIGHT-NET). *The International Journal of Cardiovascular Imaging*.

[44] D’Andrea A, Stanziola AA, Saggar R, Saggar R, Sperlongano S, Conte M (2019). Right Ventricular Functional Reserve in Early-Stage Idiopathic Pulmonary Fibrosis: An Exercise Two-Dimensional Speckle Tracking Doppler Echocardiography Study. *Chest*.

[45] Ferrara F, Gargani L, Naeije R, Rudski L, Armstrong WF, Wierzbowska-Drabik K (2021). Feasibility of semi-recumbent bicycle exercise Doppler echocardiography for the evaluation of the right heart and pulmonary circulation unit in different clinical conditions: the RIGHT heart international NETwork (RIGHT-NET). *The International Journal of Cardiovascular Imaging*.

[46] Gargani L, Pugliese NR, De Biase N, Mazzola M, Agoston G, Arcopinto M (2023). Exercise Stress Echocardiography of the Right Ventricle and Pulmonary Circulation. *Journal of the American College of Cardiology*.

[47] Baptista R, Serra S, Martins R, Teixeira R, Castro G, Salvador MJ (2016). Exercise echocardiography for the assessment of pulmonary hypertension in systemic sclerosis: a systematic review. *Arthritis Research & Therapy*.

[48] Spieker M, Lagarden H, Sidabras J, Veulemans V, Christian L, Bejinariu A (2024). Prevalence, mechanisms, and prognostic impact of dynamic mitral regurgitation assessed by isometric handgrip exercise. *European Heart Journal. Cardiovascular Imaging*.

[49] Suzuki K, Akashi YJ, Manabe M, Mizukoshi K, Kamijima R, Kou S (2013). Simple exercise echocardiography using a Master’s two-step test for early detection of pulmonary arterial hypertension. *Journal of Cardiology*.

